# Complete Genome Sequence of Porcine Circovirus Strain 102 with a Novel Mutation, Isolated from Hunan Province, China

**DOI:** 10.1128/genomeA.00411-15

**Published:** 2015-05-14

**Authors:** Zhe Zhu, Naidong Wang, Yang Zhan, Zhanfeng Wang, Aibing Wang, Zhibang Deng, Yi Yang

**Affiliations:** College of Veterinary Medicine, Hunan Agricultural University, Changsha, China

## Abstract

Porcine circovirus 2 (PCV2) strain 102 belongs to the PCV2b-1C subtype, and its sole structural protein (Cap) exhibits high homology with that of other PCV2b isolates reported in South Korea, China, and the United States. Strain 102 contains a new mutation (R37H) in the domain of the nuclear localization signal (NLS) of the Cap.

## GENOME ANNOUNCEMENT

Porcine circovirus 2 (PCV2) is a nonenveloped, icosahedral, single-stranded, circular DNA virus and belongs to the *Circoviridae* family (1, 2). PCV2 is considered to be one of the major pathogens responsible for porcine circovirus-associated diseases (PCVAD) (3–7). The genome of PCV2 (1,767 or 1,768 bp) contains three major open reading frames (ORF1, ORF2, and ORF3), with ORF1 encoding the enzymes (Rep and Rep′) involved in viral DNA replication, ORF2 encoding the PCV2 structural protein Cap, and ORF3 encoding the apoptosis-related protein (6). Previous investigations suggest that PCV2 has an evolutionary rate of 1.2 × 10^-3^ nucleotide substitutions per site every year (8), and PCV2b is one of the predominant genotypes in current pig populations (9–11).

In this study, various tissue samples (from the spleen, lymph node, liver, and lung) from postweaning multisystemic wasting syndrome (PMWS) pigs were collected in a field in Yiyang, Hunan Province, China. The complete genome of PCV2 strain 102 was amplified by PCR (2720 thermal cycler; Applied Biosystems). Subsequently, PCR products were cloned into the pSP-72 cloning vector (Promega, Madison, WI, USA) and sequenced for double-stranded DNA. The resultant sequences were assembled and analyzed using DNAStar.

The complete genome sequence of strain 102 consists of 1,767 nucleotides (nt), with a G+C content of 48.33%. It shares the highest sequence homology (99.9%) with another PCV2 strain, Han8 (GenBank accession no. JQ181600). Phylogenetic analysis suggests strain 102 can be clustered with PCV2b-1C. ORF1 of the strain consists of 945 nt, encoding 314 amino acid (aa) residues of Rep; ORF2 consists of 705 nt, encoding 235 aa residues of Cap, which shares high amino acid homology with that of PCV2b strains discovered in South Korea (GenBank accession no. KJ133547 and KJ437506), the United States (GenBank accession no. JX535296 and JX535297), and China (GenBank accession no. HM038030 and HM038017), with the exception that the Cap of strain 102 has a unique point mutation (R37H) in the nuclear localization signal (NLS). ORF3 of strain 102 consists of 315 nt, encoding an apoptosis-related protein containing 105 aa residues.

Recently, a series of mutated strains of PCV2b were discovered worldwide (12–16). Some amino acid mutations have been found in the Caps of these strains; specifically, an extra lysine (K) is present at the end of the Caps in some of the new emerging mutants and is thought to elevate PCV2 virulence (12, 16). As a consequence of the mutations in PCV2, vaccination failure in the field has been reported in the United States and South Korea (13, 14). Compared to the strain with accession no. KJ133547 found in South Korea, strain 102 has a mutation of arginine to histidine at position 37 in the Cap NLS. The same mutation in the NLS has also been found in another PCV2 strain we isolated (GenBank accession no. KJ867553). Thus, strain 102 is a candidate mutant strain behind the vaccination failures in field studies. Although it is unclear whether the new mutation has functional biological or antigenic significance, this finding facilitates the analysis of the PCV2 genomics and antigenicity.

### Nucleotide sequence accession number. 

The complete genome sequence of strain 102 has been submitted to GenBank under the accession no. KP112484.
